# Comparison of polystyrene and hydrogel microcarriers for optical imaging of adherent cells

**DOI:** 10.1117/1.JBO.29.S2.S22708

**Published:** 2024-06-13

**Authors:** Oscar R. Benavides, Berkley P. White, Holly C. Gibbs, Roland Kaunas, Carl A. Gregory, Kristen C. Maitland, Alex J. Walsh

**Affiliations:** a Texas A&M University, Department of Biomedical Engineering, College Station, Texas, United States; b Texas A&M University, Microscopy and Imaging Center, College Station, Texas, United States; c Texas A&M Health Science Center, School of Medicine, Bryan, Texas, United States

**Keywords:** light-sheet tomography, microscopy, elastic scattering, three-dimensional cell culture, microcarriers

## Abstract

**Significance:**

The ability to observe and monitor cell density and morphology has been imperative for assessing the health of a cell culture and for producing high quality, high yield cell cultures for decades. Microcarrier-based cultures, used for large-scale cellular expansion processes, are not compatible with traditional visualization-based methods, such as widefield microscopy, due to their thickness and material composition.

**Aim:**

Here, we assess the optical imaging compatibilities of commercial polystyrene microcarriers versus custom-fabricated gelatin methacryloyl (gelMA) microcarriers for non-destructive and non-invasive visualization of the entire microcarrier surface, direct cell enumeration, and sub-cellular visualization of mesenchymal stem/stromal cells.

**Approach:**

Mie scattering and wavefront error simulations of the polystyrene and gelMA microcarriers were performed to assess the potential for elastic scattering-based imaging of adherent cells. A Zeiss Z.1 light-sheet microscope was adapted to perform light-sheet tomography using label-free elastic scattering contrast from planar side illumination to achieve optical sectioning and permit non-invasive and non-destructive, *in toto*, three-dimensional, high-resolution visualization of cells cultured on microcarriers.

**Results:**

The polystyrene microcarrier prevents visualization of cells on the distal half of the microcarrier using either fluorescence or elastic scattering contrast, whereas the gelMA microcarrier allows for high fidelity visualization of cell morphology and quantification of cell density using light-sheet fluorescence microscopy and tomography.

**Conclusions:**

The combination of optical-quality gelMA microcarriers and label-free light-sheet tomography will facilitate enhanced control of bioreactor-microcarrier cell culture processes.

## 1 Introduction

There has been great interest in developing therapeutics based on mesenchymal stem cells (MSCs) due to their anti-inflammatory and immunomodulatory properties.^1^^–^^3^ However, the use of cell-based therapies, or cytotherapies, in the clinic will require manufacturing batches of trillions of cells and the development of efficient large-scale stem cell manufacturing protocols to meet this demand.^4^ One promising large-scale expansion strategy is to culture MSCs on spherical microcarriers maintained in suspension,^5^^–^^7^ which can produce a higher cell yield than monolayer cultures while maintaining the viability, identity, and functional potential of MSCs.^8^^–^^12^ Microcarriers can be made from different materials including solids such as polystyrene and glass or hydrogels such as gelatin methacryloyl (gelMA).^13^^,^^14^ Polystyrene microcarriers are readily available but are non-degradable and require a filtration step to harvest cells after expansion; gelMA microcarriers, however, are biodegradable, which improves cell harvest and manufacturing scalability.

Cell culture health is typically assessed through visualization-based methods; unfortunately, standard widefield microscopy techniques used to observe monolayer cultures do not readily translate to three-dimensional (3D) microcarrier-based cultures due to the microcarrier thickness (100+ μm) and high refractive index of traditional microcarrier materials (polystyrene, glass).^15^^,^^16^ Furthermore, the assessment of cell culture parameters, such as cell density and morphology, requires a 3D imaging technique to capture the entire microcarrier surface.^17^ Due to the large diameters and relatively high refractive indices of microcarriers, the high-resolution visualization of cells along the entire microcarrier surface requires a volumetric optical imaging technique capable of optical sectioning to reconstruct the 3D object.^13^^,^^18^^–^^22^

Light-sheet fluorescence microscopy (LSFM) is a fast and photo-gentle camera-based imaging method that utilizes fluorescence contrast for image formation. Unfortunately, LSFM requires exogenous labels for fluorescence contrast or higher laser power for autofluorescence imaging, both of which can be toxic for live cells.^23^ Light-sheet tomography, however, uses elastic scattering for contrast and allows for rapid and direct 3D visualization of individual cells on microcarriers without the need for contrast agents. Light-sheet tomography thus enables robust non-invasive and non-destructive monitoring of cell culture attributes to assess culture quality.^24^ Because elastic scattering contrast relies on refractive-index heterogeneities in the sample, microcarriers comprised of materials with high refractive indices may scatter light intensely and preclude the visualization of weak-scattering cells on their surface.

**Fig. 1:**
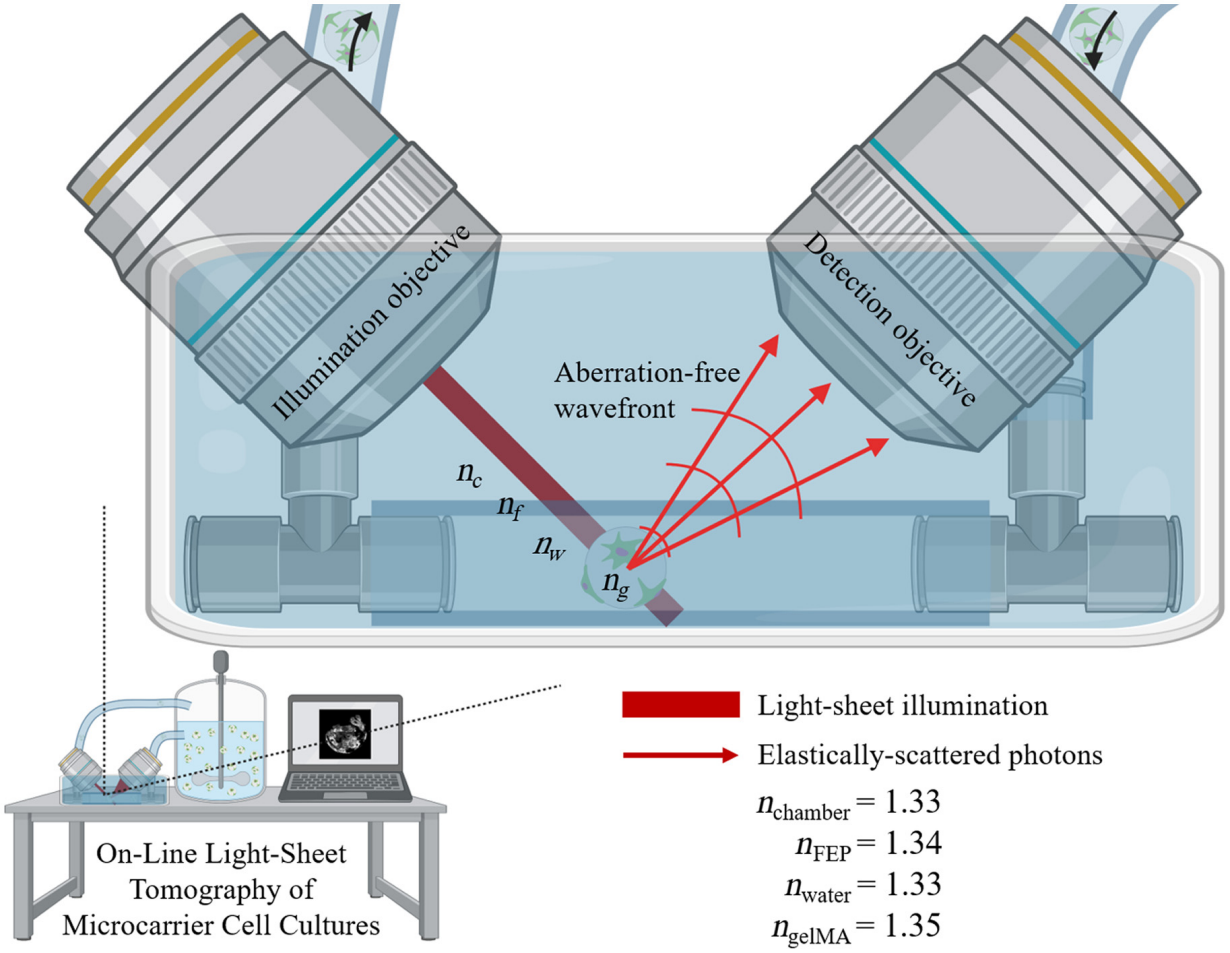
Illustration of on-line light-sheet tomography system as a process analytical technology for monitoring microcarrier-based cell cultures. The illumination light-sheet propagates along a refractive index-matched optical path to generate elastically scattered photons for image contrast. The emitted wavefront is minimally aberrated permitting diffraction-limited imaging through the gelMA microcarrier.

Here, we compare the light-sheet tomography imaging compatibilities of microcarriers comprised of two different materials with different index of refraction values. Commercial polystyrene microcarriers (n=1.59) are compared with custom-fabricated gelMA microcarriers (n=1.34) and assessed as microcarrier substrates for light-sheet tomography visualization and enumeration of MSCs. Mie scattering and wavefront map simulations of polystyrene and gelMA microcarriers are performed to describe the angular scattering intensity distributions and microcarrier-induced wavefront errors, respectively. The optical properties of polystyrene and gelMA microcarriers are then directly compared for compatibility with light-sheet imaging for the visualization of cells and subcellular features and the enumeration of adherent cells from fluorescence light-sheet and elastic scattering light-sheet images. This work motivates the use of hydrogel microcarriers and light-sheet tomography for the non-destructive characterization of microcarrier-based cell cultures (Fig. 1).

## 2 Methods

### 2.1 gelMA Microcarrier Synthesis

The gelMA microcarriers were produced following a previously published protocol.^13^ Briefly, gelMA was first synthesized by reacting type A porcine gelatin (Sigma) and methacrylic anhydride at 0.6 mL methacrylic anhydride per gram gelatin in phosphate buffered saline (PBS) for 1 h at 50°C. The reaction was quenched with 40°C PBS then dialyzed through a 12 to 14 kDA nitrocellulose membrane against deionized water at 40°C for 7 days. Then, gelMA and photoinitiator lithium phenyl-2,4,6-trimethylbenzoylphosphinate were dissolved at a final concentration of 7.5% (w/v) and 10 mM, respectively, in PBS at 50°C. A custom polydimethylsiloxane (Sylgard 184) microfluidic device was created and bound to a 2″ × 3″ glass slide after 1 min of oxygen plasma treatment and cured overnight at 85°C. Syringe pumps (Cole Parmer) were used to flow the gelMA solution and fluorinated oil (Novec 7500) through the microfluidic device at 2 and 4  mL/h, respectively. The gelMA and oil droplets were then exposed to 365 nm light ($75\;\mathrm{mW}/{\mathrm{cm}}^{2}$) for 50 s to polymerize the microcarriers. The gelMA microcarriers were collected from the residual oil solution via centrifugation at 3000 g for 3 min on a glycerol bed. Finally, the microcarriers were recovered from the glycerol bed and washed with PBS and stored at 4°C.

### 2.2 Bone Marrow-Derived MSCs Cultured on Polystyrene Microcarriers

Bone marrow-derived human MSCs (BM-hMSCs)^25^ transfected with red fluorescence protein (dsRed) were expanded on polystyrene microcarriers (P-221, Pall-Solohill) in a 10 mL rotating wall vessel (RWV) bioreactor (Synthecon) and fixed with neutral buffered formalin. The microcarriers have a manufacture-provided size range of 125 to 212  μm. First, lentiviral transduction was used to make cells express dsRed (ThermoFisher, Waltham, Massachusetts). Then, BM-hMSCs were initially expanded in low-density monolayer cell culture in complete culture medium (CCM) [α-minimum essential medium, 20% (w/v) fetal bovine serum, 2 mM L-glutamine, 100  U/mL penicillin, and 100  μg/mL streptomycin] to obtain the required cell numbers.^25^ Finally, collagen I-coated polystyrene microspheres and BM-hMSCs were incubated at $1000\text{ cells}/{\mathrm{cm}}^{2}$ at 37°C for 2 h in 10 mL CCM with orbital mixing at 30 revolutions per minute (RPM). Cells were fixed with 4% paraformaldehyde (PFA) and stored in PBS at a concentration of 3 mg particles/mL PBS. Prior to imaging, fixed microcarrier-cell samples were incubated with a 5  μM DRAQ-5 DNA buffer at 37°C for 30 min with agitation, then rinsed with PBS, and embedded in 1% agarose gel for imaging.

### 2.3 ihMSCs Cultured on gelMA Microcarriers

Passage 4 ihMSCs were first expanded in a low-density monolayer cell culture in CCM to obtain the required cell numbers. As previously described elsewhere,^24^ the ihMSCs^26^ were cultured in an RWV bioreactor (RCCS-8DQ bioreactor, Synthecon) at 24 RPM on custom 120±6.2  μm diameter gelMA microcarriers at $1000\text{ cells}/{\mathrm{cm}}^{2}$.^13^ Specimens were suspended in 1 mM concentration of CellTracker Green (CTG) (Thermo Scientific) for 45 min, then fixed with 4% PFA, and stored in PBS. Prior to imaging, samples were incubated with a 5  μM DRAQ-5 (Thermo Scientific) DNA staining buffer at 37°C for 30 min with agitation and then rinsed with PBS. CTG permits the visualization of the cell cytoplasm and quantification of cell morphology. DRAQ-5 is used for cell nuclei visualization and direct cell enumeration.

### 2.4 Light-Sheet Fluorescence Microscopy

*In situ* fluorescence imaging of cells attached to microcarriers was performed on a Zeiss Z.1 Lightsheet microscope (LSM) using a 20× 1.0 NA (water) detection objective lens and 10× 0.2 NA illumination objective lenses (air). BM-hMSCs were illuminated with 561 (DsRed) and 638 nm (DRAQ-5). ihMSCs were illuminated with 488 (CTG) and 638 nm (DRAQ-5). The emission filters used were 505 to 545 nm (CTG), 600 to 600 nm (DsRed), and 660+ nm (DRAQ-5). A variable zoom of 1.16× was used for an effective magnification of 23.2×, and the voxel size was 0.2  μm×0.2  μm×0.45  μm to satisfy Nyquist sampling requirements. Even illumination of the entire microcarrier surface was achieved using 5% to 15% power with dual-sided objective illumination with pivot scanning and online maximum intensity fusion. Z-stacks were acquired at 50 frames per second (FPS) using pco.edge 5.5M sCMOS cameras.

### 2.5 Light-Sheet Tomography

Light-sheet tomography based on elastic scattering contrast was performed on the Z.1 LSM with the same illumination and detection optics. The laser blocking filter and emission filters were removed from the optical path between the detection objective lens and the camera to allow scattered light to enter the detection path. Samples were illuminated using the 638 nm laser at 0.1% power, which was sufficient to fill the dynamic range of the camera at 50 FPS. Imaging was performed with dual-sided objective illumination with pivot scanning and online maximum intensity fusion.

### 2.6 Mie Scattering Simulations

The open-source Mie scattering software MiePlot (v4.6.21) was used to simulate the angular distribution of Mie scattering intensity.^27^ The software accepts refractive index (n) inputs for the immersion medium and particle, particle size, illumination wavelength, and illumination polarization. The polystyrene particles were modeled as a sphere with a 165  μm diameter and n equal to 1.59,^28^ and the gelMA microcarriers were spheres with a 120  μm diameter and n equal to 1.35.^24^ Cells were simulated to have a diameter of 10  μm and n equal to 1.45.^29^^,^^30^ The combined water and agarose immersion media was modeled with n equal to 1.34. The refractive indices of the agarose immersion media and gelMA microcarriers were measured using a handheld refractometer (KRUSS Optronic).

### 2.7 Wavefront Map Simulations

The ray-tracing software ZEMAX was used to simulate the widefield detection arm of the LSM imaging through either the polystyrene or gelMA microcarrier.

The wavefront map simulation was used to calculate the microcarrier-induced wavefront error, represented by peak-valley and root mean square (RMS) error. The objective and tube lenses were simulated as paraxial lenses of focal length equal to 9 and 180 mm, respectively, to represent the 20× 1.0W Olympus objective and Olympus tube lens. The imaging medium was set with n equal to 1.33 to simulate the use of the water-dipping objective lens. The cell, or object plane, is situated on the distal hemisphere of the microcarrier, which is placed at the front focal plane of the objective lens. The gelMA microcarrier was simulated as a 120  μm sphere of n equal to 1.34; the polystyrene microcarrier was simulated as a 165  μm sphere of n equal to 1.59; in addition, the gelMA microcarrier was simulated as 165  μm and the polystyrene as 120  μm to compare wavefront errors induced by varying the particle size.

### 2.8 Data Analysis

The open-source image analysis software ImageJ/FIJI was used to generate 2D intensity projections of the 3D microcarrier fluorescence and scattering volumes. ImageJ was also used to characterize fluorescence signal attenuation with increasing depths by creating an orthogonal projection of the microcarriers and extracting intensity profiles at varying depths of the microcarrier. MATLAB 2020b was used for statistical analysis of the normalized maximum intensity depth profiles of the two microcarriers and the simulated 100% confluent microcarrier. The optical surface area per slice of a sphere was calculated by integrating the sphere surface area using the following equation:

$$A=4\pi r^{2}\int_{0}^{\pi /2}\;\sin\;\theta \;d\theta .$$

## 3 Results

### 3.1 Mie Scattering Simulations

**Fig. 2:**
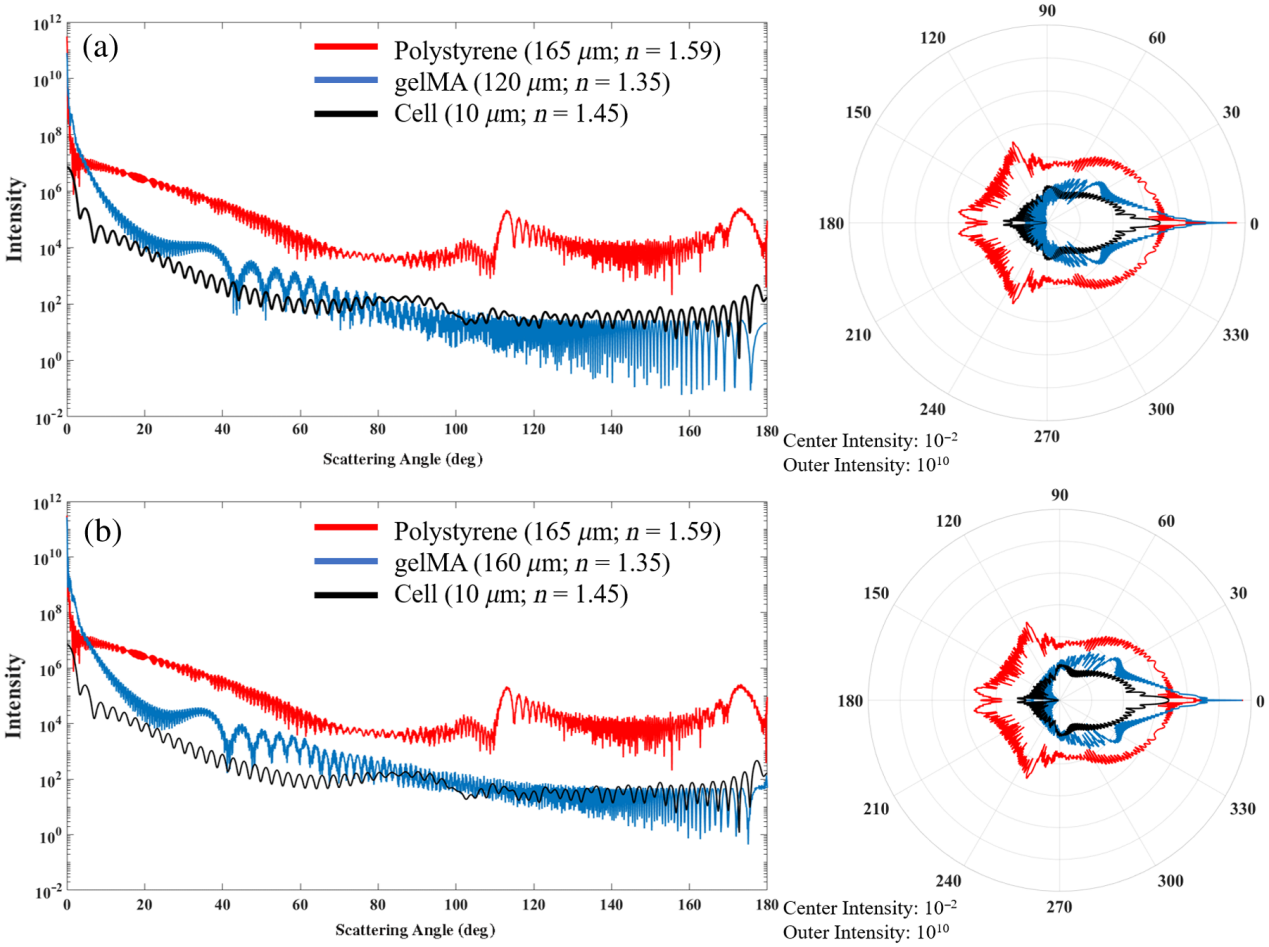
Mie scattering intensity simulations of polystyrene and gelMA microcarriers and single cells on (a) rectangular and polar plot showing the polystyrene microcarrier orthogonal scatters at much higher intensities, which inhibits the visualization of cells along the surface using LSM. (b) The gelMA microcarrier was simulated as the same diameter (165  μm) as the polystyrene microcarrier to decouple the scattering intensity from microcarrier size.

To assess the potential for elastic scattering-based contrast imaging of cells expanded on spherical microcarriers, Mie simulations for the two microcarriers were performed using MiePlot. The angular distributions of Mie scattering intensity for the two microcarriers and a cell were simulated using an unpolarized light source [Fig. 2(a)]. In addition, the gelMA microcarrier was simulated as a 165  μm diameter to match the diameter of and compare it to the polystyrene microcarrier [Fig. 2(b)].

These simulations show that the polystyrene microcarriers scatter light much more intensely than the hydrogel microcarriers and the cells. This phenomenon is due to the refractive index mismatch of the surrounding water immersion media and the microcarrier. The Mie simulations predict the scattering intensity to be two to four orders of magnitude greater for polystyrene than gelMA microcarriers. The cells, with an overall refractive index between water and polystyrene, scatter light near the same intensity range as the hydrogel microcarriers. This phenomenon permits the visualization of cells along the entire gelMA microcarrier surface using both fluorescence and elastic scattering contrast.

### 3.2 Wavefront Map Simulations

**Fig. 3:**
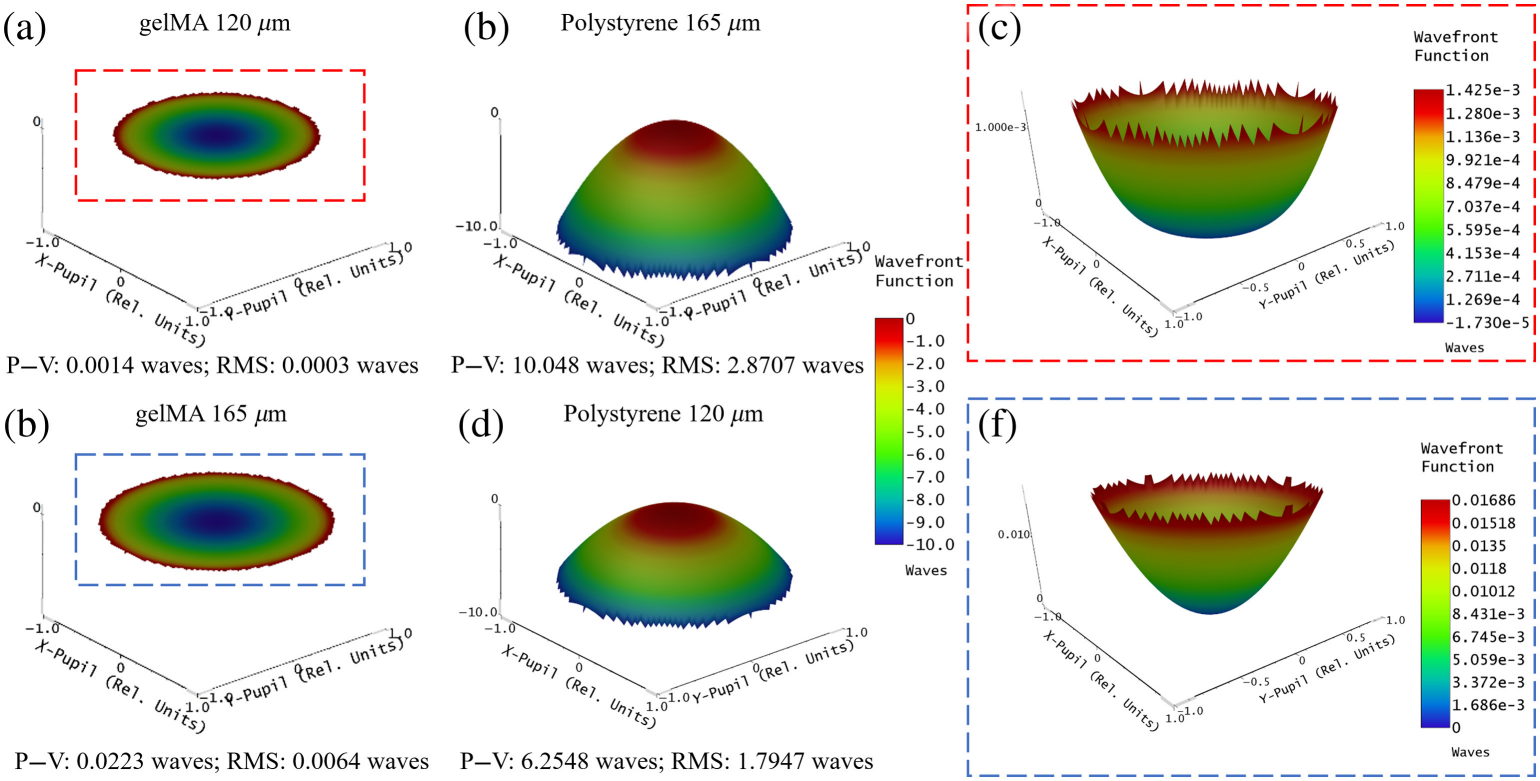
ZEMAX simulations of wavefront errors introduced by 120 and 165  μm diameter gelMA and polystyrene microcarriers. gelMA microcarriers of (a) 120  μm diameter introduce an RMS OPD of 0.0003 waves, and increasing the microcarrier diameter to (b) 165  μm introduces RMS OPD of 0.0064 waves. Polystyrene microcarriers of (c) 165  μm diameter introduce severe aberrations, resulting in RMS OPD of 2.8707 waves, and decreasing the microcarrier diameter to (d) 120  μm only slightly improves the image quality, resulting in RMS OPD of 1.7947 waves. Zoomed-in view of (e) 120 and (f) 165  μm gelMA microcarrier minimally aberrated wavefronts.

To assess the optical aberrations induced by imaging through the microcarrier, the light-sheet detection arm was simulated in ZEMAX with the distal microcarrier surface at the objective lens front focal plane. The wavefront map simulation was used to quantify the microcarrier-induced peak–valley (P−V) and RMS wavefront errors, which are key indicators of optical system performance. These simulations show that the gelMA microcarrier causes less optical path differences (OPD) than the polystyrene microcarrier (Fig. 3). In fact, the gelMA RMS error (0.0003 waves) is <1/4λ, which is the Rayleigh criterion that describes the threshold for diffraction-limited imaging performance [Fig. 3(a)]. Increasing the gelMA microcarrier diameter from 120 to 165  μm increases the RMS wavefront error to 0.0064 waves [Fig. 3(b)]; however, this is still less than the error (2.8707 waves) introduced by the 160  μm polystyrene microcarrier [Fig. 3(c)]. When the polystyrene microcarrier is simulated as a 120  μm diameter, the RMS wavefront error (1.7947 waves) is still greater than that of the 120 and 165  μm gelMA microcarriers. These simulations illustrate that the custom-fabricated gelMA microcarrier induces fewer optical imaging aberrations compared with commercially available polystyrene microcarriers.

### 3.3 Microcarrier Screening for Label-Free Elastic Scattering-Based Visualization

**Fig. 4:**
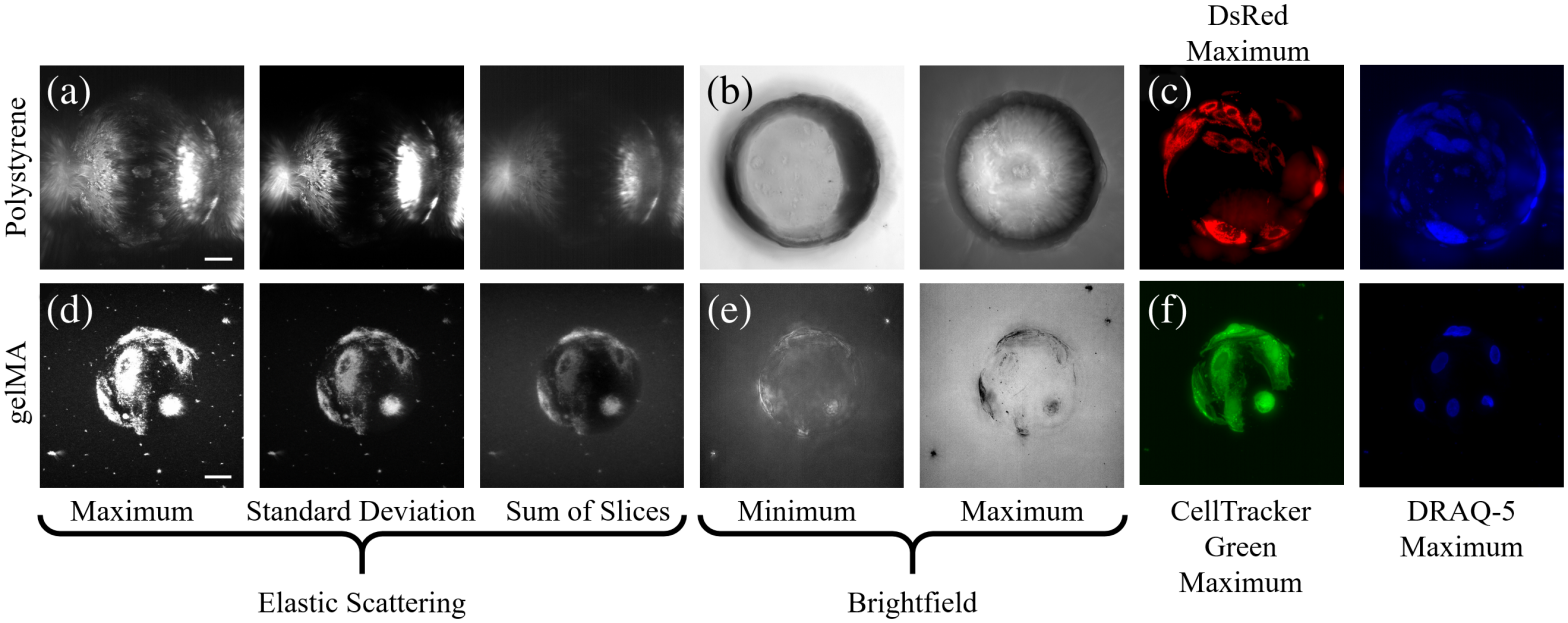
The gelMA microcarriers are superior for the label-free visualization of the entire culture surface area. Label-free intensity-based projections of the polystyrene microcarrier using (a) elastic scattering and (b) brightfield contrast provide limited visualization of cells on the microcarrier surface compared with (c) exogenous fluorescence contrast. The gelMA microcarrier permits the use of label-free (d) elastic scattering and (e) brightfield, and (f) exogenous fluorescence contrast for the visualization of cells along the entire microcarrier surface.

The polystyrene microcarrier scatters much more intensely than cells cultured on the microcarrier surface. This causes the cell signal to be obscured by the polystyrene microcarrier scattering [Fig. 4(a)]. Only cells situated on the polystyrene microcarrier equator and proximal hemisphere can be visualized using brightfield transmitted illumination [Fig. 4(b)]. This necessitates the use of fluorescence contrast to visualize and distinguish cells from the polystyrene microcarrier surface [Fig. 4(c)]. The gelMA microcarrier, conversely, scatters at intensities near or lower than cells cultured on the gelMA microcarrier surface, which permits the sub-cellular visualization of cells along the entire 3D surface using light-sheet tomography [Fig. 4(d)]. Standard deviation and sum of slices contrast enhancement projections even permit the simultaneous visualization of the microcarrier, cells, and surrounding agarose. Brightfield illumination can also be used to visualize and identify cells from the gelMA microcarrier surface [Fig. 4(e)]. Populated microcarriers can be distinguished from empty microcarriers using brightfield illumination to visualize textural features on the microcarrier surface. Fluorescence contrast similarly provides an equivalent view of cells cultured on the gelMA microcarrier surface [Fig. 4(f)]. These exemplary volumetric projections illustrate the compatibility of the hydrogel microcarrier for the label-free visualization of cells along the entire microcarrier surface.

### 3.4 Microcarrier Screening for Direct Fluorescence-Based Cell Enumeration

**Fig. 5:**
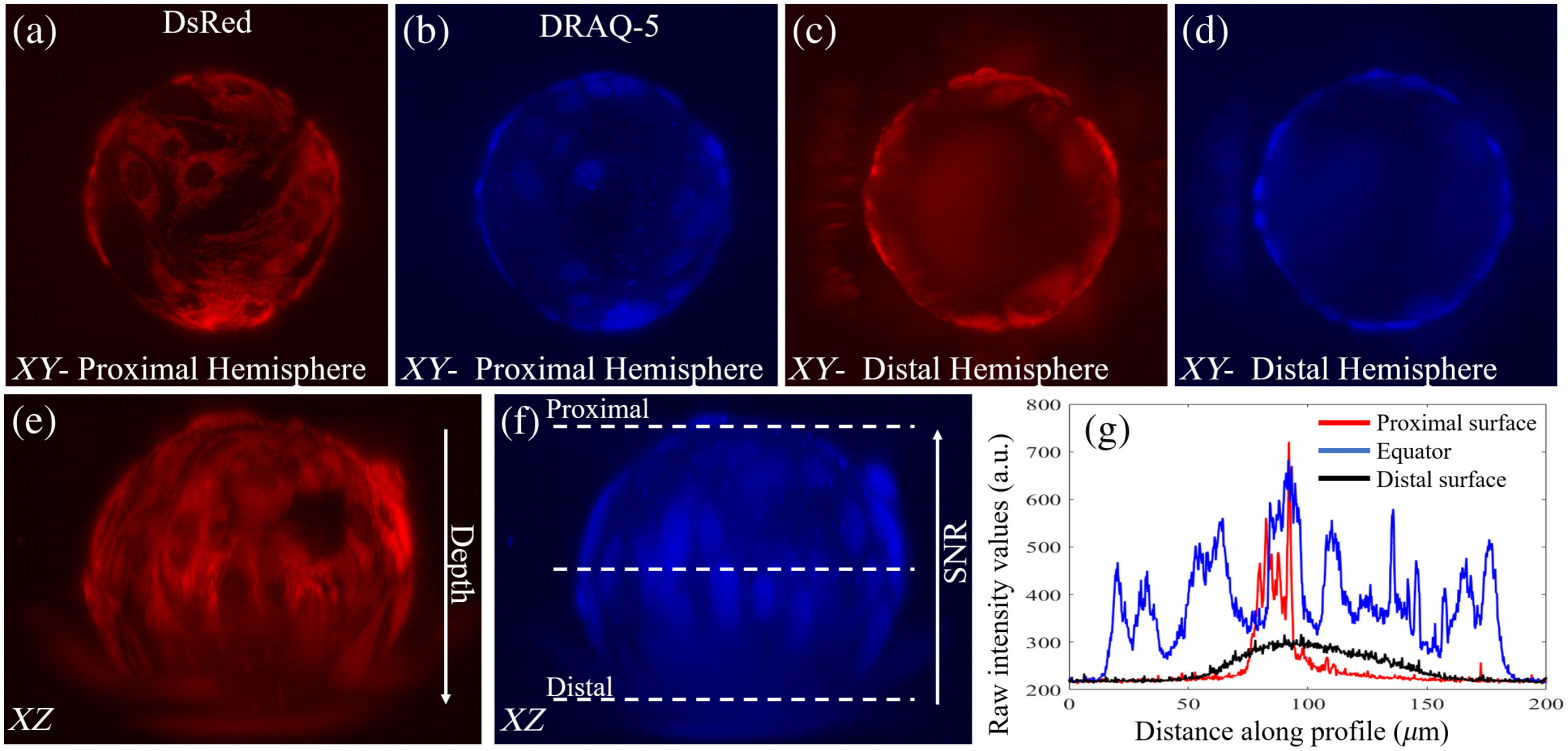
Polystyrene microcarriers limit visualization to the proximal microcarrier surface. Maximum (a) dsRed and (b) DRAQ-5 intensity projections of the proximal microcarrier surface. (c) dsRed and d) DRAQ-5 maximum intensity projections of the distal microcarrier surface. (e) dsRed and (f) DRAQ-5 signal intensity is (g) attenuated beyond the microcarrier equator. Line projections of fluorescence intensity in (g) correspond to dashed lines in (f).

**Fig. 6:**
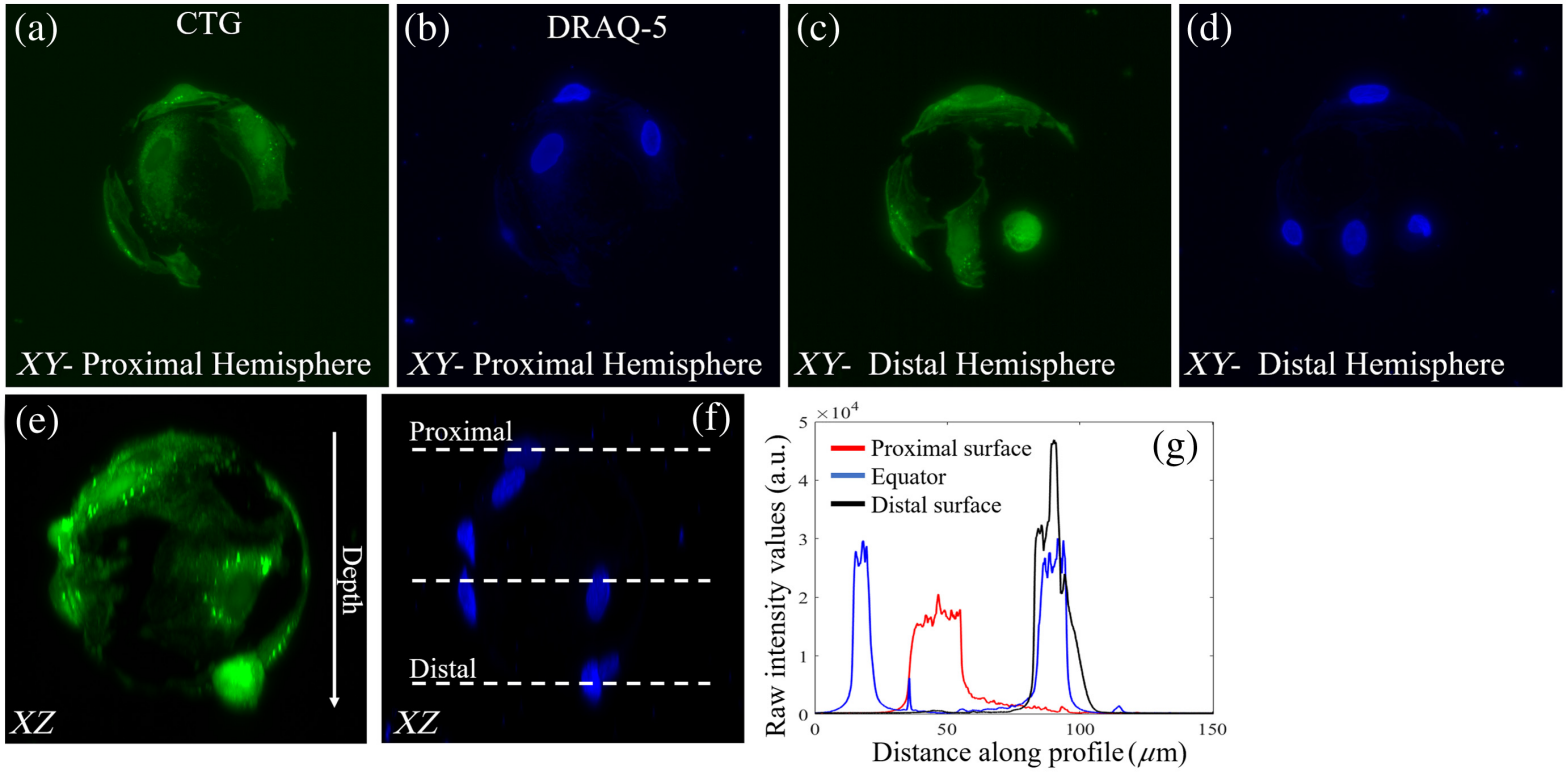
gelMA microcarriers enable high resolution visualization of the entire microcarrier surface. Maximum (a) CTG and (b) DRAQ-5 intensity projections of the proximal microcarrier surface. (c) CTG and (d) DRAQ-5 maximum intensity projections of the distal microcarrier surface. (e) CTG and (f) DRAQ-5 signal intensity is (g) maintained throughout the entire depth of the microcarrier. Line projections of fluorescence intensity in (g) correspond to dashed lines in (f).

To evaluate the optical imaging performance of the different microcarriers, fixed MSCs attached to commercially available 165  μm diameter polystyrene and 120  μm diameter custom-fabricated hydrogel microcarriers were imaged with an LSFM. Representative data of DsRed and DRAQ-5 labeled BM-hMSCs on polystyrene and CTG and DRAQ-5 labeled ihMSCs on gelMA microcarriers are shown in Figs. 5 and 6.

The polystyrene microcarrier inhibits the visualization of cells on the distal half of the microcarrier equator [Figs. 5(a)–5(f)]. There are 30 cell nuclei identified via DRAQ-5 on the microcarrier equator and proximal surface, only one or two additional cell nuclei can be identified on the distal surface near the equator, and no cell nuclei are resolved in the center of the distal microcarrier hemisphere [Figs. 5(b) and 5(d)]. This is because the fluorescence signal intensity is attenuated with depth, and the ability to visualize, resolve, and enumerate cells beyond the microcarrier equator is severely hindered [Figs. 5(e) and 5(f)]. Cells on the distal half of the microcarrier are illuminated, but the polystyrene microcarrier blurs these slices in the stack and the ability to resolve individual nuclei is eliminated [Fig. 5(g)]. A scattering artifact caused by the microcarrier can be seen on the left hemisphere of the microcarrier in Figs. 5(c) and 5(d).

The hydrogel-based gelMA microcarriers, compared with commonly used polystyrene microcarriers, enable high fidelity visualization and semi-automatic direct enumeration of cells along the entire microcarrier surface [Figs. 6(a)–6(d)]. There are seven cell nuclei resolved from the DRAQ-5 contrast [Fig. 6(f)]. The fluorescence intensity is preserved along the entire depth of the microcarrier, and cell nuclei can be visualized on both the proximal and distal hemisphere of the microcarrier [Figs. 6(e)–6(g)].

### 3.5 Depth-Dependent Fluorescence Signal Attenuation

**Fig. 7:**
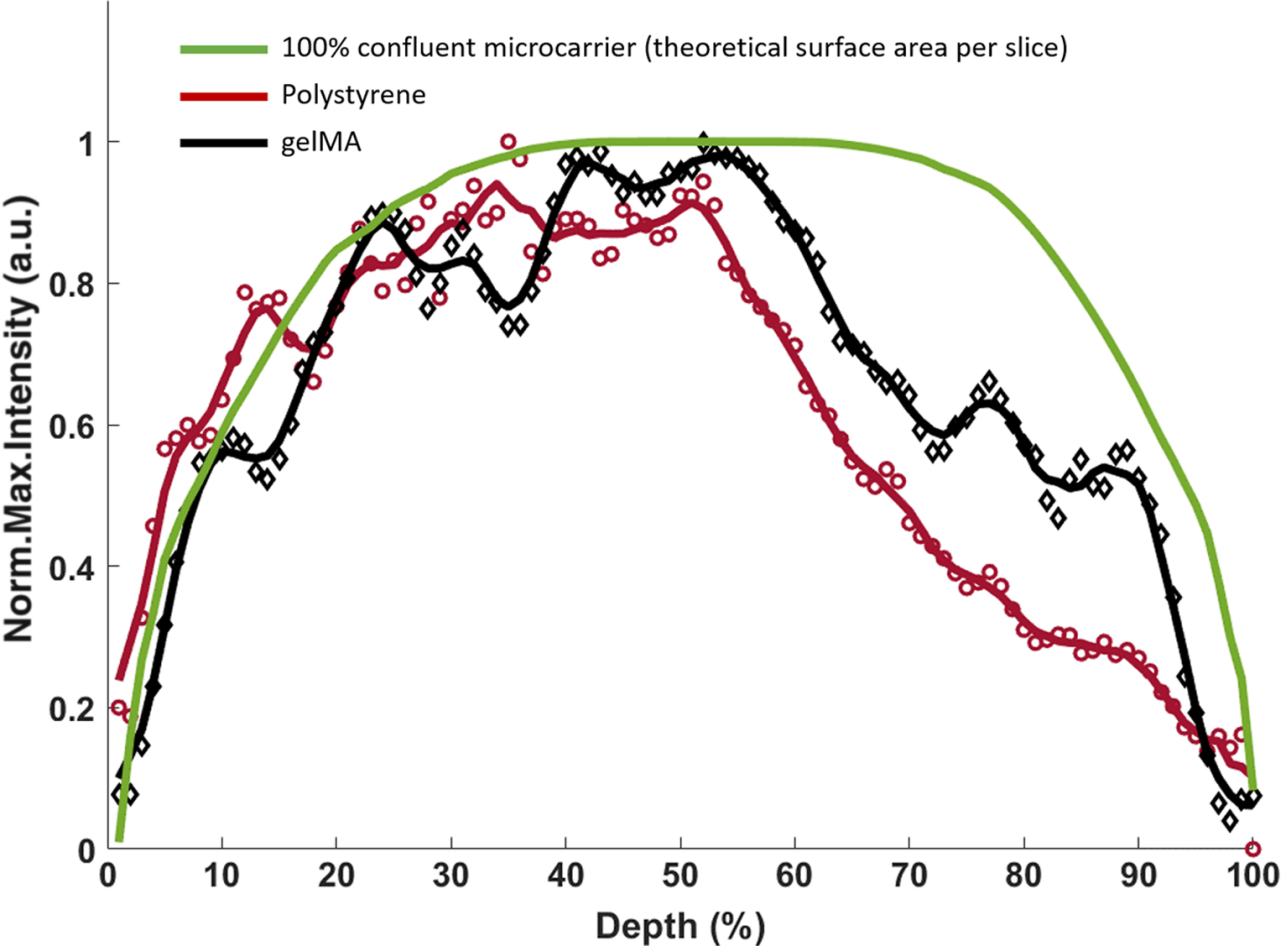
gelMA microcarriers have superior volumetric optical imaging capabilities compared polystyrene microcarriers. Normalized average maximum intensity-depth profile of gelMA (n=17) and polystyrene (n=31) microcarriers showing the large fluorescence intensity attenuation that occurs on the distal half of the polystyrene microcarriers compared with gelMA microcarriers. A five-point sliding average filter was applied to both microcarrier profiles (solid lines). A 100% confluent microcarrier is simulated using the theoretical surface area per optical slice.

To compare the depth-dependent fluorescence signal intensity attenuation caused by the microcarrier material, the maximum DRAQ-5 intensity per frame per microcarrier profile is averaged, normalized, and plotted for both microcarrier materials; in addition, the theoretical illuminated microcarrier surface area is plotted as a reference for a 100% confluent microcarrier. Only single microcarriers with two or more cells and >20% CTG confluency were included to select for moderate and highly confluent single microcarriers and to avoid including aggregated microcarriers in the analysis. The gelMA microcarriers have a more symmetrical profile than the polystyrene microcarriers, and these data show that the gelMA microcarriers cause less depth-dependent fluorescence signal intensity attenuation than the polystyrene microcarriers (Fig. 7). A two-sample Kolmogorov–Smirnov test was performed to quantitatively assess the similarity of the depth-intensity profiles between the polystyrene and gelMA microcarriers. The gelMA and polystyrene microcarriers showed a statistically significant difference from each other (P=0.0314). The gelMA microcarrier profile did not show statistically significant difference from the 100% confluent microcarrier (P=0.0994), whereas the difference between the polystyrene and 100% confluent microcarrier profiles was found to be statistically significant (P=0.0082). Data were considered to be significant if the P values were <0.05. Both microcarrier material intensity profiles show a decrease in intensity and deviation from the theoretical confluent microcarrier beginning at around 55% depth; this is an interesting phenomenon that may be due to the spherical nature of the microcarriers or a sample size artifact. Mie scattering of spherical objects is well known to have both broad and sharp intensity oscillations termed wiggles and ripples, respectively, that are due to the spherical shape of the scattering object;^31^^,^^32^ the depth-dependent fluorescence intensity oscillations seen here could similarly be related to the spherical nature of the microcarriers.

## 4 Discussion

The FDA has encouraged therapeutic manufacturers to deploy process analytical technologies (PATs) as a means to monitor and control a manufacturing process by gathering (near) real-time insight into critical attributes of the therapeutic product that can be incorporated into a control strategy to assure product quality.^33^ The combination of optically compatible microcarriers with rapid, label-free, high-resolution imaging and robust image analysis-based characterization of adherent cell cultures will greatly enhance the ability to monitor large-scale expansion of cells for cell-based therapies.

In this study, we compared the optical imaging compatibilities of commercial polystyrene microcarriers versus custom-fabricated gelMA microcarriers for non-destructive and non-invasive visualization of the entire microcarrier surface, direct cell enumeration, and sub-cellular visualization of MSCs using Mie scattering and wavefront error simulations and empirical light-sheet imaging data. The Mie scattering simulations explain previous results using reflectance confocal microscopy and light-sheet tomography for scattering-based volumetric imaging of microcarrier cell cultures;^13^^,^^24^ the polystyrene microcarrier scattering intensity obscures the cell scattering signal, and fluorescence modalities are unable to visualize the distal hemisphere of the polystyrene microcarrier. Image formation from the distal microcarrier hemisphere requires photons scattered by cells to reach the camera, but these signal-contributing photons are likely to be deflected and/or absorbed by the polystyrene microcarrier before they reach the detector. Light-sheet imaging is traditionally performed with de-coupled and orthogonal illumination and detection paths, and the detection objective used here has a 97.5 deg angular aperture. It is apparent from the Mie scattering plots that the polystyrene microcarrier scatters more intensely than the gelMA microcarrier in the detection objective lens angular aperture range, as well as in the forward direction, which is relevant for transmitted illumination imaging schemes.

The wavefront error simulations further demonstrate that the gelMA microcarrier induces smaller wavefront aberrations compared with polystyrene microcarriers of the same diameter; this permits diffraction-limited, high-resolution imaging through the entire gelMA microcarrier volume and the visualization of cells along the entire microcarrier surface. The polystyrene microcarrier causes a greater wavefront error due to the refractive index mismatch between itself and the water imaging medium, whereas the gelMA microcarrier has a minimal refractive index mismatch with water. The ZEMAX simulation was performed using sequential mode and does not consider scattering from the microcarriers, which would further reduce the image quality from polystyrene microcarriers.

The imaging data illustrate the limited optical imaging compatibility of commonly employed polystyrene microcarriers for *in toto* quantitative monitoring of ihMSC growth on microcarriers. The polystyrene microcarrier does not permit the visualization of the entire 3D surface due to the absorption of light and its refractive index mismatch with the imaging medium, which causes optical aberrations and high-intensity scattering; thus, the observation of cells is limited to the proximal hemisphere of the polystyrene microcarrier. The custom gelMA microcarriers are optically transparent with a refractive index close to water, which allows for the label-free visualization of the entire microcarrier core and surface. These experimental findings are supported by both the Mie scattering and wavefront error simulations, which show the value in refractive index matching of the microcarrier for label-free scattering-based 3D imaging of MSCs. In addition, cell nuclei fluorescently labeled with DRAQ-5 situated anywhere on the microcarrier surface can be visualized with a high spatial resolution and SNR as there is minimal refractive index mismatch between the gelMA microcarrier material and the water imaging medium that would cause optical aberrations. Cell enumeration is, therefore, not limited to the superficial half of the microcarrier surface and does not require the membrane lysis or detachment of cells from the microcarrier surface.

Volumetric optical imaging enables high fidelity visualization of the entire microcarrier surface. Coupled with hydrogel microcarriers, cells can be visualized using fluorescence and elastic scattering contrast. The use of light-sheet tomography and gelMA microcarriers permits faster acquisition and visualization of adherent cells using multiple orders of magnitude less power than is required for fluorescence contrast. The angular scattering intensity distribution profiles of the microcarriers were simulated to assess the potential for elastic scattering-based imaging of the cells cultured on the microcarrier surface. The wavefront error simulations further motivate refractive index matching of the microcarrier material and imaging medium for high-quality imaging data of adherent cells on spherical microcarriers. In addition, the optical imaging compatibilities of polystyrene and gelMA microcarriers were quantitatively compared and show a significant difference in the ability to visualize and characterize the entire microcarrier surface.

## 5 Conclusion

Optically transparent microcarriers composed of gelMA enable non-invasive and non-destructive *in toto* quantification of cell density and microcarrier surface area confluency. Conversely, the polystyrene microcarrier causes greater scattering of illumination light than the hydrogel microcarrier, and this prevents the visualization of cells on the distal microcarrier hemisphere using either fluorescence or elastic scattering contrast. The gelMA microcarriers, when combined with light-sheet imaging, permit the high-fidelity visualization of cells in 3D and quantification of critical cell culture parameters, such as cell density and morphology. Label-free light-sheet tomography combined with the gelMA microcarriers enables rapid, non-destructive, *in toto* imaging of adherent cells. This avoids the use of exogenous fluorescent labels, which are destructive to the cells and thus decrease the yield of the cell culture process. Label-free contrast is, therefore, essential for in- or on-line monitoring of cell cultures not only to preserve the sterility of the culture and minimize the waste of valuable resources and product but also for rapid results to enable real-time decision making in the upstream manufacturing process. The combination of optical-quality gelMA microcarriers and label-free light-sheet tomography will aid in the development of PATs for cytotherapy manufacturing and facilitate the enhanced control of the bioreactor-microcarrier cell culture processes.

## Data Availability

All relevant data, materials, and software code used in this research are available upon request from the corresponding author.
